# Smoking behavior detection algorithm based on YOLOv8-MNC

**DOI:** 10.3389/fncom.2023.1243779

**Published:** 2023-08-24

**Authors:** Zhong Wang, Lanfang Lei, Peibei Shi

**Affiliations:** ^1^School of Artificial Intelligence and Big Data, Hefei University, Hefei, China; ^2^School of Computer Science and Technology, Hefei Normal University, Hefei, China

**Keywords:** smoking behavior detection, YOLOv8, MHSA, NWD, CARAFE

## Abstract

**Introduction:**

The detection of smoking behavior is an emerging field faced with challenges in identifying small, frequently occluded objects like cigarette butts using existing deep learning technologies. Such challenges have led to unsatisfactory detection accuracy and poor model robustness.

**Methods:**

To overcome these issues, this paper introduces a novel smoking detection algorithm, YOLOv8-MNC, which builds on the YOLOv8 network and includes a specialized layer for small target detection. The YOLOv8-MNC algorithm employs three key strategies: (1) It utilizes NWD Loss to mitigate the effects of minor deviations in object positions on IoU, thereby enhancing training accuracy; (2) It incorporates the Multi-head Self-Attention Mechanism (MHSA) to bolster the network’s global feature learning capacity; and (3) It implements the lightweight general up-sampling operator CARAFE, in place of conventional nearest-neighbor interpolation up-sampling modules, minimizing feature information loss during the up-sampling process.

**Results:**

Experimental results from a customized smoking behavior dataset demonstrate significant improvement in detection accuracy. The YOLOv8-MNC model achieved a detection accuracy of 85.887%, signifying a remarkable increase of 5.7% in the mean Average Precision (mAP@0.5) when compared to the previous algorithm.

**Discussion:**

The YOLOv8-MNC algorithm represents a valuable step forward in resolving existing problems in smoking behavior detection. Its enhanced performance in both detection accuracy and robustness indicates potential applicability in related fields, thus illustrating a meaningful advancement in the sphere of smoking behavior detection. Future efforts will focus on refining this technique and exploring its application in broader contexts.

## 1. Introduction

Smoking behavior detection has gradually attracted more and more attention in recent years. With the increase in public health awareness and a deeper understanding of the harms of smoking, more and more individuals and organizations are beginning to focus on how to effectively identify and prevent smoking behaviors (Ashare et al., 2021). Smoking behavior detection involves using computer vision technology to automatically recognize and locate human smoking behaviors in images or videos, thereby monitoring and controlling smoking scenarios. This technology can be applied in practical applications such as public places, factories, schools, etc. (Liu X. et al., 2023), helping to enforce smoking bans, strengthen the management of smoking areas, protect the environment, and reduce pollution (Shi et al., 2023). Smoking behavior detection generally relies on deep learning models for training and inference, effectively reducing the manual cost of smoking detection and improving detection accuracy and efficiency (Tian et al., 2023). Generally speaking, these algorithms can be divided into two branches. One is sensor-based detection methods, such as inhalation sensor-based detection (Yu et al., 2022), lip sensor-based detection (Imtiaz et al., 2019), and hand sensor-based detection (Skinner et al., 2017). These methods face several challenges. They involve high computational load and complex manual feature extraction. Additionally, they exhibit weak feature representation capability and poor model generalization. As a result, solving smoking detection problems across various scenarios becomes quite challenging. The other is using convolutional neural network algorithms to extract features from images, thereby recognizing smoking targets. Common target detection frameworks include YOLO (Jiang P. et al., 2022), Faster R-CNN (Li et al., 2015), SSD (Leibe et al., 2016), and Heterogeneous Networks of Graph Neural Networks (GNNs) (Wang Y. et al., 2022). These algorithms learn and train from a large amount of smoking image data to achieve efficient and accurate target detection.

**FIGURE 1:**
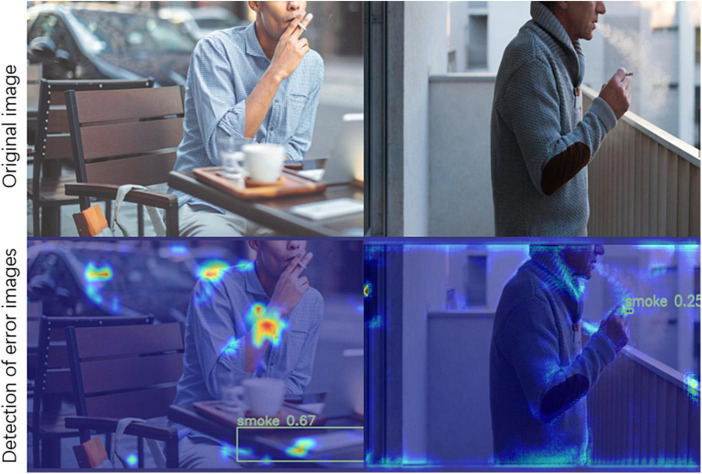
Example diagram of smoking error detection.

Despite the significant improvements in smoking detection due to deep convolutional networks, there are still some challenges. First, smoking detection needs to consider the influence of the surrounding environment on smoking images, such as intense illumination, complex backgrounds, and occlusion. These factors may cause biases or misjudgments in the model. Secondly, smoking behavior exhibits certain diversified characteristics. For instance, when recognizing cigarettes, information regarding shape and size needs to be noted. These characteristics also increase the difficulty in algorithm training and practical application. Finally, smoking detection requires the use of high-precision sensors and cameras, which can increase the system development and maintenance costs. Also, in large-scale applications, one may need to consider hardware resource limitations, as well as other constraints. Consequently, in practical applications, due to the impact of the above factors, there may be problems such as false detection, missed detection, and a low detection rate, as shown in Figure 1. These issues may affect the accuracy and reliability of the detection results. Therefore, it is necessary to take corresponding measures to address these problems and improve the accuracy and reliability of the detection.

To address these issues, this paper proposes the YOLOv8-MNC algorithm, which is an improvement on the faster and more accurate YOLOv8, and applies it to smoking behavior detection. The main contributions are as follows:

1. Incorporating NWD Loss to mitigate the sensitivity of IoU to minor object position deviations, thereby enhancing the training accuracy.
2. Incorporating the Multi-head Self-attention Mechanism (MHSA) to boost the global feature learning ability of the target object in the convolution network.
3. Utilizing the lightweight general up-sampling operator CARAFE to replace the original nearest-neighbor interpolation up-sampling module, thereby reducing the loss of feature information during the up-sampling process.
4. Proposing the smoking behavior detection algorithm YOLOv8-MNC, based on YOLOv8. On our custom dataset, the detection accuracy during training reached 85.887%, with a mean Average Precision (mAP) that was 5.7% higher compared to the YOLOv8 algorithm.

The rest of this paper is structured as follows: section “2. Related works” provides a review of relevant works in the field of smoking behavior detection. Section “3. Materials and methods” delves into the enhanced YOLOv8-MNC algorithm framework and explicates the specifics of its implementation. In section “4. Experimental results,” we assess the performance of our proposed method through a series of experimental tests. Finally, the paper concludes with a summary and outlines potential future directions.

## 2. Related works

Presently, methods for detecting smoking behavior primarily comprise traditional and computer vision-based approaches. Traditional methods employ smoke sensors to detect cigarette smoke, thereby identifying smoking behavior. Wu and Chen (2011) proposed a system for smoking behavior detection through facial analysis, which accurately and rapidly discerns whether individuals in images are smoking. Iwamoto et al. (2010) introduced a smoke detection method based on image sequences, utilizing convolutional neural networks (CNNs) to process continuous video frames and detect the presence of smoke. Ali et al. (2012) presented an automated system named mPuff for detecting inhalations of cigarette smoke from respiratory measurements. With the rapid development of computer vision and deep learning, an increasing number of smoking detection algorithms based on object detection have been proposed. Adebowale and Lwin (2019) put forward a deep learning algorithm architecture based on convolutional neural networks (CNNs) and long short-term memory (LSTM) networks, for detecting smoking behavior from respiratory signals. Rentao et al. (2019) proposed an indoor smoking behavior detection approach that adds a small-scale detection layer to the traditional YOLOv3-tiny network. Poonam et al. (2019) used the Faster RCNN algorithm for cigarette target detection, demonstrating robustness to lighting and deformations. Zhang et al. (2018) proposed a new smoking detection algorithm based on CNNs, which differentiates between non-smokers and smokers by recognizing the position and posture of smokers in photos or videos through feature extraction and classifiers. Liao and Zou (2020) proposed using the DarkNet53 as the backbone feature extraction network and decoding the YOLOv3 model through Bounding Box after outputting the feature map to detect smoking behavior within the monitored area. Jiang X. et al. (2022) introduced a smoking behavior detection method based on the YOLOv5 network, which captures images using a camera and recognizes and locates smokers in the images using the YOLOv5 algorithm. Wang Z. et al. (2022) proposed an improved YOLOv5-based architecture with the addition of new data enhancement techniques such as RandomErasing and GaussianBlur to enhance the robustness of the model for real-time smoke detection. Hu et al. (2022) introduced a fast detection algorithm for forest fire smoke using MVMNet, which is designed to extract and classify image features for smoke detection. Liu et al. (2022) proposed an IoT security solution named Adaptive multi-channel Bayesian graph attention network (AMGBA), aiming to address security issues in the Internet of Things. Xu et al. (2023) introduced a bimodal emotion recognition algorithm using mixed features of audio and speech context. Liu et al. (2020) presented a method for ESD soft fault detection based on Linux kernel function call analysis. Liu et al. (2018) proposed a method for heat exchange analysis in deep-sea spectral detection systems based on ROV, including detailed modeling. Liu Z. et al. (2023) discussed a graph structure learning method of EGNN, focusing on its application in graph neural networks.

In the field of object detection, the challenge of accurately identifying small targets, such as cigarettes in smoking detection, has been a persistent issue. These small objects often occupy only a minor portion of the entire image, leading to difficulties in extracting precise position and feature information. Existing methods have approached this problem through various techniques, but limitations remain. Deep learning algorithms for small target detection commonly adopt methods that focus on multi-scale features, contextual information, and loss functions. In terms of multi-scale features, Lin et al. (2017a) utilized FPN to fuse high-resolution and high semantic information for the Faster RCNN, achieving a 17.8% average precision for small target detection. Liu et al. (2019) improved scale invariance by suppressing inconsistencies in spatial-temporal feature fusion, achieving a 43.9% AP on the YOLOv3 and MS COCO dataset. Gong et al. (2021) introduced a “fusion factor” to control information flow between deep and shallow network layers, enhancing small target learning efficiency. Regarding contextual information, Leng et al. (2021) proposed an internal-external network-based detector (ENe) that leverages target appearance and context, enhancing feature extraction, localization, and classification. Guan et al. (2018) proposed the Semantic Context Aware Network (SCAN), utilizing pyramid pooling to fuse multi-level context, thereby improving small target detection. In the realm of loss functions, Wang J. et al. (2021) used the Wasserstein distance to measure bounding box similarity, replacing standard IoU, and demonstrated that using NWD in R-CNN increases network convergence time. Xu et al. (2022) proposed a Gaussian Receptive Field based Label Assignment (RFLA) strategy, enhancing tiny target detection and achieving a 24.8% average precision on the AI-TOD dataset. Akyon et al. (2022) presented SAHI (Slicing Aided Hyper Inference), an open-source framework for small target detection in high-resolution images. Zhang et al. (2020) introduced the MultiResolution Attention Extractor (MRAE) to learn attention weights across different layers, fusing features with weighted sums, and improving small target detection precision without the need for GAN or data preprocessing.

YOLO is currently the most popular real-time object detector, encompassing versions such as YOLOv5 (Zhu et al., 2021), YOLOv7 (Wang C. Y. et al., 2022), and YOLOv8. For example, YOLOv5 focuses on optimizing speed and efficiency, YOLOv7 introduces new features for better handling of small objects, and YOLOv8 further refines the architecture for improved accuracy and robustness. Compared to the previous version YOLOv4 (Bochkovskiy et al., 2020), both YOLOv5 and YOLOv7 have made improvements in speed and accuracy. However, YOLOv5 exhibits some drawbacks, such as deficiencies in small target detection and the need for improvements in dense target detection. YOLOv7 is also limited by training data, model structure, and hyperparameters, leading to performance degradation in certain situations. YOLOv8, an anchor-less object detection algorithm, incorporates new network structures like PAN-FPN and Decoupled-Head, but it still struggles with small object recognition in complex scenes. For instance, during feature extraction by the neural network, small targets can be misled by large ones, and the features extracted from deep layers may lack sufficient small target information. This deficiency causes the algorithm to ignore small targets during the learning process, leading to poor detection performance. Compared to normal-sized objects, small-sized ones are more likely to be overlapped by other objects and partially obscured by objects of other sizes, making them difficult to distinguish and locate in an image. Existing methods have approached this problem through techniques such as multi-scale training, specialized loss functions, feature fusion, and attention mechanisms.

To address these issues, we propose a new detection algorithm, YOLOv8-MNC, based on the YOLOv8 algorithm. It significantly enhances the detection performance for small-sized objects while maintaining the detection effectiveness for normal-sized ones.

## 3. Materials and methods

### 3.1. Overview of the YOLOv8-MNC

**FIGURE 2:**
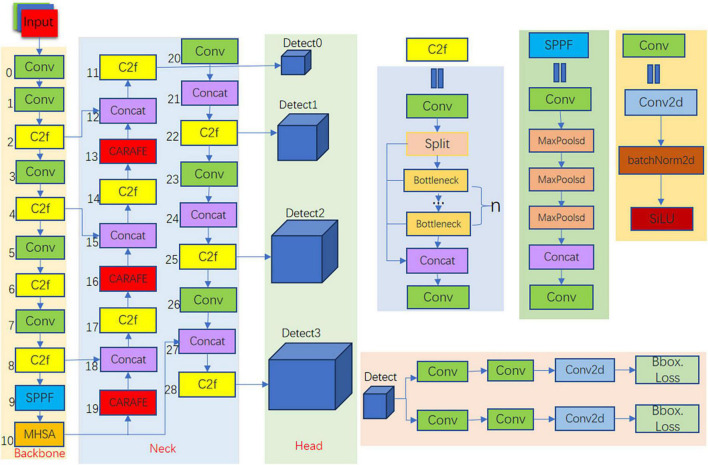
YOLOV8-MNC structure.

YOLOv8 is the latest iteration of the YOLO series of detection models, renowned for their joint detection and segmentation capabilities. We have enhanced it and introduced it into the field of smoking detection. The architecture of our YOLOv8-MNC detector is shown in Figure 2. It consists of three parts: the backbone, the head, and the neck. YOLOv8-MNC is based on the CSP concept and improves YOLOv5 by replacing the C3 module with the C2f module. Compared with the C3 module, the C2f module can better capture feature information and improve detection accuracy. At the same time, the CSP concept can effectively reduce the amount of calculation and improve the running speed of the model. The C2f module borrows the ELAN idea from YOLOv7, combining C3 and ELAN to form the C2f module, allowing it to maintain a lightweight design while obtaining richer gradient flow information. In the penultimate layer of the backbone, we still use the most popular SPPF module, passing three 5 × 5 Maxpools of different sizes in succession, and then concatenating each layer. This not only ensures the accuracy of objects at different scales but also ensures the lightweight nature of the objects. We added this module to the SPPF module to help the convolutional network learn the global characteristics of the target object. The MHSA attention mechanism can adaptively adjust the weights between different features, so as to better capture the global information of the target object and improve the performance of the model. In the neck part, the feature fusion method we use is PAN-FPN, which enhances the fusion and utilization of feature layer information at different scales. We used three lightweight upsampling operators called CARAFE and multiple C2f modules, along with a decoupled head structure, to form the neck module. The idea of decoupling the head in YOLOX is used in the last part of the neck. It combines confidence and regression boxes to improve training accuracy. The upsampling operator CARAFE replaces the original nearest neighbor interpolation, reducing the loss of feature information during the upsampling process.

### 3.2. Improvement measures

#### 3.2.1. MHSA module network structure

**FIGURE 3:**
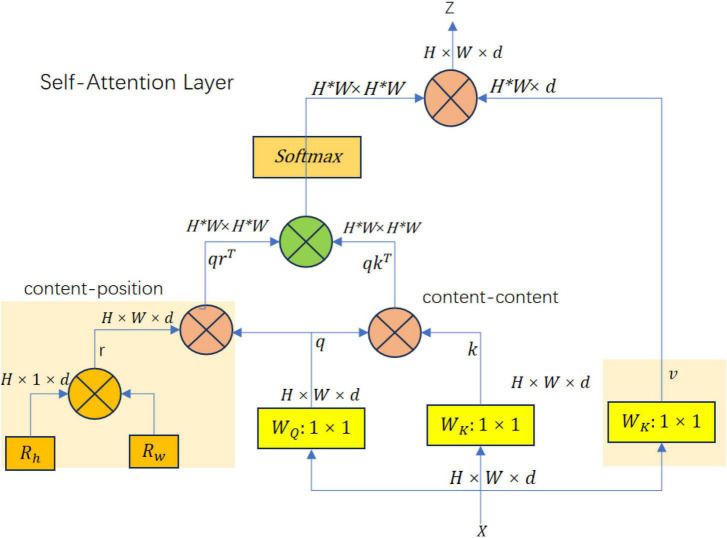
MHSA module network structure.

With the wide application of Transformer in the field of Computer Vision (CV), models such as ViT (Wang Y. et al., 2021) for image classification tasks, DETR (Carion et al., 2020) and Deformable DETR (Zhu et al., 2020) for object detection tasks are all designed based on the Transformer concept. In the attention mechanism, Srinivas et al. (2021) proposed the Bottleneck Transformer module, which designed the Multi-Head Self-Attention Layer (MHSA) based on the Non-local idea. This structure reduces the number of parameters while optimizing the backbone feature extraction network. The structure of the multi-head self-attention layer is shown in Figure 3. For the current input, feature *Z*^*H* × *W* × *d*^ three different weight matrices *W*_K_, *W*_Q_, *W*_V_ are first initialized. These initialized matrices representing query, key, and value are used to compute the representation of the input features, respectively. These representations are used in the self-attention mechanism to compute attention weights, and the input features are weighted and averaged to generate attention-enhanced feature representations. After calculations, q, w, and v, three vectors of dim, are obtained. Unlike the multi-head self-attention mechanism, MHSA uses a similar spatial attention mechanism to handle position encoding. *R*_h_ and *R*_w_ are two learnable vectors, which can be used as attention vector representations in the horizontal and vertical spatial directions. The sum of these two vectors gives a two-dimensional spatial encoding r. After the vector dot product calculation between r and q, spatial similarity is obtained. The content similarity is obtained after the vector dot product calculation between q and k. After adding the two, it is converted into attention weights through Softmax, and then the dot product calculation with v yields the attention-enhanced feature representation.

Spatial similarity is derived from the dot product between the relative position encoding vector r and the query vector q, capturing the geometric structure within the data. Content similarity, on the other hand, is obtained from the dot product between the query vector q and the key vector k, focusing on semantic relationships. Together, these similarities provide a comprehensive understanding of both the geometric and semantic aspects of the input, enhancing the model’s ability to recognize complex patterns in tasks such as object detection and image classification. The multi-head self-attention layer directly replaces the 3 × 3 convolution in the last residual block of ResNet, and the output feature can be used in various downstream tasks. It is a good way to enhance the model’s ability to model input features and the ability to perceive the relationship between different positions. The introduction of relative position encoding in the MHSA layer not only considers content information, but also considers the relative distance between features at different positions, which can effectively correlate the information and position perception between objects.

#### 3.2.2. NWD

In YOLOv8, the Anchor-Free method is used for object detection. The core idea is to divide the input image into S × S grid units, each referred to as a “Cell.” Within each Cell, B bounding boxes (abbreviated as BOBox) are predicted. Each bounding box contains a center point coordinate (CP) and a width and height. These bounding boxes can cover the entire input image, thereby detecting all possible targets. Compared to traditional detection methods, the Anchor-Free method does not require predefined anchor boxes but predicts the target’s position and category directly on the feature map.

In the entire Anchor mechanism, Intersection over Union (IoU) is an essential metric for determining positive and negative labels based on thresholds or for excluding bounding boxes with high redundancy. In the training process, a large number of anchor boxes are generated. To obtain the anchor box’s target category and the real box’s offset to the anchor box, the calculation of IoU is utilized to acquire the anchor box’s label. Similarly, in the prediction phase, a single target will generate multiple similar prediction boxes, thereby significantly increasing the computational load significantly. Hence, IoU is used as a threshold, adopting non-maximum suppression to get the optimal prediction box.

**FIGURE 4:**
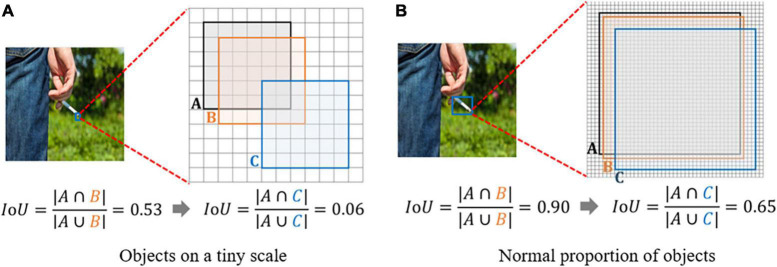
**(A)** Objects on a tiny scale. **(B)** Normal proportion of objects.

Small targets in an image often contain only a few pixels, lacking substantial appearance information and details. The Intersection over Union (IoU) and its extensions are highly sensitive to the positional deviation of small targets; even minor shifts can cause a significant drop in IoU, leading to errors in label allocation. When applied to algorithms based on the Anchor mechanism, this sensitivity can adversely affect detection performance. As illustrated in Figure 4, minor positional deviations can lead to considerable changes in IoU. Given the critical role of IoU in label allocation, even a slight numerical difference might cause what should be allocated to positive samples to be assigned to negative ones. Moreover, if the scale of some targets is too small, the overlap between the anchor box and the real box may never meet the threshold, resulting in an average number of positive samples allocated by the actual box of less than one.

IoU only works when bounding boxes overlap. Hence, GIoU (Lin et al., 2017b) was proposed to solve this problem by adding a penalty term. But when two bounding boxes contain each other, GIoU degrades to IoU. Subsequently, DIoU (Zheng et al., 2019) and CIoU (Zheng et al., 2020) were proposed to overcome these issues. However, GIoU, DIoU, and CIoU are all extensions of IoU, commonly used in loss functions. They still exhibit sensitivity to positional deviations of small target objects in label allocation. To overcome these shortcomings, this paper adds NWD (Wang J. et al., 2021) to the CIoU loss function, with both components accounting for half of the total loss function. The primary step of NWD is to model the bounding box as a two-dimensional Gaussian distribution, then use NWD to measure the similarity of the derived Gaussian distributions. NWD can measure distribution similarity even in non-overlapping cases, and it is insensitive to the scale of the target. It is particularly suitable for measuring the similarity of small target objects.

For small target objects, since most real objects are unlikely to be standard rectangles, the bounding boxes often carry some background information. The information of the target object and the background information are concentrated at the center point and the boundary of the bounding box, respectively. Therefore, when creating a two-dimensional Gaussian distribution for the bounding box, the center pixel of the bounding box can be set as the highest weight, which then gradually decreases from the center point to the boundary. For a horizontal bounding box R, μ *and* ∑ represent the mean vector and covariance matrix of the Gaussian distribution, which can be fitted into a two-dimensional Gaussian distribution *N*(μ, ∑), where:


(1) $$R=\left(c_{x},c_{y},w,h\right),\mu =\left[\begin{array}{c} c_{x}\\ c_{y} \end{array}\right],\sum =\left(\begin{array}{cc} \frac{W^{2}}{4} & 0\\ 0 & \frac{h^{2}}{4} \end{array}\right)$$

In this way, the similarity between bounding boxes is transformed into the distance between Gaussian distributions, where (*c*_x,_ , *c*_y_) are the center coordinates of the bounding box, and w and h are the width and height. The Wasserstein distance is used to calculate the distribution distance. The second order Wasserstein distance between different bounding boxes μ_1_ and μ_2_ is as follows:


(2) $$W_{2}^{2}\,\left(\mu_{1},\mu_{2}\right)={||m_{1}-m_{2}||}_{2}^{2}$$

$$+T\,r\,\left(\sum_{1}+\sum_{2}-2\,{\left(\sum_{2}^{1/2}\sum_{1}\sum_{2}^{1/2}\right)}^{1/2}\right)$$

where


(3) $$\mu_{1}=N\,\left(m_{1},\sum_{1}\right),\mu_{2}=N\,\left(m_{2},\sum_{2}\right)$$

Using Gaussian distributions *N*_*1*_ and *N*_*2*_, where *N*_*1*_ represents bounding box *N*_*2*_ represents bounding box B, the formula can finally be simplified as:


(4) $$W_{2}^{2}\,\left(N_{1},N_{2}\right)$$

$$={||\left({\left[c_{1\,x},c_{1\,y},\frac{w_{1}}{2},\frac{h_{1}}{2}\right]}^{T},{\left[c_{2\,x},c_{2\,y},\frac{w_{2}}{2},\frac{h_{2}}{2}\right]}^{T}\right)||}_{2}^{2}$$

Where


(5) $$A=\left(c_{1\,x},c_{1\,y},w_{1},h_{1}\right),B=\left(c_{2\,x},c_{2\,y},w_{2},h_{2}\right)$$

As $W_{2}^{2}\,\left(N_{1},N_{2}\right)$ functions as a unit of distance rather than a similarity measure, and IoU operates as a ratio bounded between 0 and 1, the necessity to normalize this distance arises. This leads to the computation of the Normalized Wasserstein Distance (NWD), which yields a standardized measure suitable for comparison. The final normalized result is NWD (Normalized Wasserstein Distance):


(6) $$W\,L\,\left(N_{1},N_{2}\right)=\exp\left(-\frac{\sqrt{W_{2}^{2}\,\left(\mu_{1},\mu_{2}\right)}}{C}\right)$$

where C is a constant set empirically, set as 12.8 in this paper.

#### 3.2.3. Lightweight upsampling operator CARAFE (content-aware ReAssembly of features)

**FIGURE 5:**
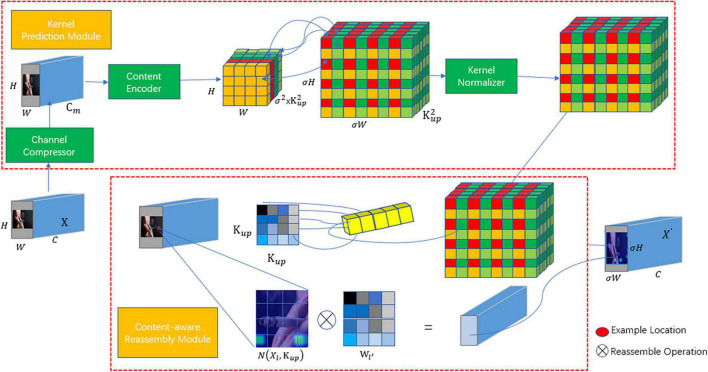
Structure of the light weight upsampling operator CARAFE.

The original YOLOv8 feature fusion network employs nearest neighbor interpolation, using the grayscale value of the closest pixel among neighboring pixels around the sampling point. This approach neglects the influence of other neighboring pixel points, and the grayscale value becomes discontinuous after resampling, leading to a loss of image quality. In contrast, the improved method, within the PAFP structure introduces the lightweight upsampling operator CARAFE (Content-Aware ReAssembly of Features) (Loy et al., 2019) to replace nearest neighbor interpolation. The CARAFE structure is mainly divided into two parts: the upsampling kernel prediction module and the feature recombination module. First, the upsampling kernel prediction module utilizes the input feature map to predict the sampling kernel. Then, it uses the predicted upsampling kernel to recombine the features and complete upsampling process. These recombined features can rectify the feature deviation that occurs during the fusion process. Characterized by low redundancy, lightweight design, rapid computation, strong feature fusion ability, and fast running speed, the CARAFE operator is a significant enhancement. By replacing the feature fusion network with the CARAFE operator, it can aggregate contextual information within a larger receptive field. This method abandons the nearest neighbor interpolation approach for samples, opting instead for a single kernel sampling method, and generates an adaptive content-aware sampling technique. The feature fusion network with the introduced CARAFE operator is depicted in Figure 5.

The CARAFE computation process can be divided into the following four parts:

(1) Channel Compression: The input H × W × C dimensional features are compressed to *H* × *W* × *C*_m_ dimensions to reduce the amount of computation in subsequent operations. Where *C*_*m*_ is the number of compressed channels, in this paper *C*_m_ is set to 64.
(2) Content Encoding and Upsampling Kernel Prediction: For the compressed feature map, an upsampling kernel of size $\sigma_{H}\times \sigma_{\text{W}}\times K_{\text{up}}^{2}$ is predicted using a convolutional layer with a convolution kernel of *K*_encoder_ × *K*_*encoder*_. Where *K*_up_ is the size of the predicted upsampling kernel, in this paper *K*_up_ is 5, *K*_encoder_ is 3.
(3) Upsampling Kernel Normalization: The predicted upsampling kernel is normalized by Softmax to make the sum of the convolution kernel weights 1.
(4) Content-Aware Feature Recombination: The predicted upsampling kernel is convolved with the input features to obtain the recombined features.

## 4. Experimental results

### 4.1. Dataset and experimental setup

**FIGURE 6:**
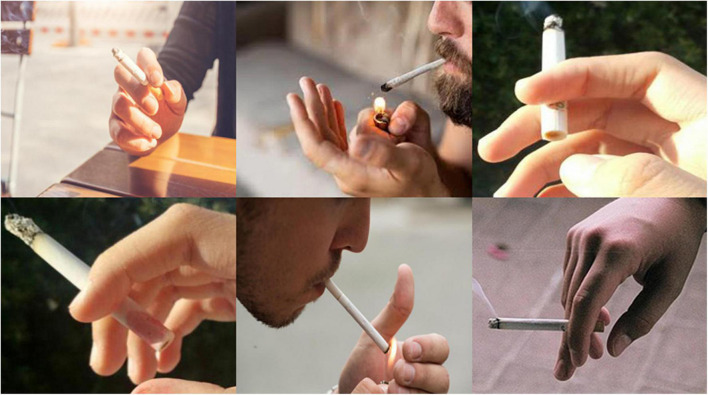
Image of the dataset.

For the specific task of smoking detection, this study relies on a self-constructed dataset, as public datasets are lacking in this domain. The dataset was assembled from smoking-related images sourced from the Internet through keyword searches and manual screening, as well as key frames extracted from recorded smoking video clips. The combined collection was then meticulously cleaned and screened to remove noise and outliers, with the aid of advanced image and video processing technologies, including deep learning-based image processing. The final dataset comprises a total of 11,629 images, all annotated using Labelimg in the PASCAL VOC format. Prior to training, the annotations were converted into the txt format required by YOLOv8, and the dataset was partitioned into training and validation sets at a 7:3 ratio. The detection task focuses solely on categorizing smoking behavior, labeled as “smoke” within the dataset. The dataset, as depicted in Figure 6, represents a comprehensive and carefully curated resource for the study’s experimental needs.

This study was conducted using the PyTorch deep learning framework, with code execution and model training carried out on the Inspur Artificial Intelligence platform server, equipped with an ASPEED Graphics Family (rev 41) graphics card. The system operates on Red Hat 4.8.5–44, utilizing Python 3.8, CUDA 11.3, and PyTorch 1.12.1 tools. Specifically, the model was trained over 500 epochs to ensure comprehensive learning, with a learning rate of 0.01 to balance convergence speed and accuracy. The Stochastic Gradient Descent (SGD) optimizer was employed to efficiently update the model parameters, making it suitable for handling the large-scale dataset.

### 4.2. Model evaluation

This paper uses precision, recall, Average Precision (AP), and Mean Average Precision (mAP) as model accuracy evaluation indicators. AP represents the area under the Precision-Recall (PR) curve, and mAP represents the average of the AP for each class. TP represents the number of correctly predicted positive samples, which reflects the performance of the model in accurately detecting positive samples. FN represents the number of positive samples that were incorrectly predicted as negative samples, revealing positive samples that the model may have missed. FP represents the number of negative samples that are incorrectly predicted as positive samples, indicating that the model may incorrectly label negative samples as positive samples. The specific formulas are as follows:


(7) $$P=\frac{T\,P}{T\,P+F\,P}$$

(8) $$R=\frac{T\,P}{T\,P+F\,N}$$

(9) $$A\,P=\frac{\sum P}{N\,u\,m\,\left(o\,b\,j\,e\,c\,t\,s\right)}$$

(10) $$m\,A\,P=\frac{\sum A\,P}{N\,u\,m\,\left(c\,l\,a\,s\,s\right)}$$

### 4.3. Experimental results

#### 4.3.1. Experimental comparison of different loss functions

To validate the effects of different loss functions, we used the YOLOv8 model as a baseline and selected CIoU (Zheng et al., 2020), SIoU (Gevorgyan, 2022), EIoU, Wise-IoU (Tong et al., 2023), Focal-EIoU (Zhang et al., 2021), and NWD (Wang J. et al., 2021) for experimental comparison. As shown in Table 1, the mAP@0.5 values for EIoU, Focal_EIoU, CIoU, Wise-IoU, SIoU, and CioU+NWD are 80.965, 81.484, 81.805, 81.883, 81.86, and 82.777, respectively. mAP@0.5 is an important indicator for evaluating the performance of the target detection model, and a higher mAP@0.5 value represents the accurate detection ability of the model for the target object. We can observe that the CioU+NWD loss function performs significantly better than other loss functions in the experiment, obtaining the highest mAP@0.5 value of 82.777. It is particularly worth noting that compared with the original CIoU, the mAP@0.5 value of CioU+NWD is increased by 1.293%. This demonstrates that the introduction of NWD effectively reduces the sensitivity to small object position deviations, and solves the localization problem of small objects while improving training accuracy. Therefore, this further validates the effectiveness of incorporating NWD into the CiOU loss function.

**TABLE 1:** Comparison of different loss functions.

Loss function	Map0.5/%
EioU	80.965
Focal-EIoU	81.484
CioU	81.805
SioU	81.86
CiOU + NWD	82.777

#### 4.3.2. Experimental comparison of different attention mechanisms

We have made improvements to the activation function in YOLOv8 by using CELU and added a small object detection layer and attention mechanism based on NWD for comparison. We selected 11 different attention mechanisms for comparison, including TripletAttention (Misra et al., 2020), CoTAttention (Li et al., 2021), ShuffleAttention (Yang, 2021), Polarized Self-Attention (Liu H. et al., 2021), GAM_Attention (Liu Y. et al., 2021), CAM_concat (Xiao et al., 2021), SKAttention (Li et al., 2019), GlobalContext (Cao et al., 2019), EffectiveSE (Lee and Park, 2019), ParNetAttention (Goyal et al., 2021), SimAM (Yang et al., 2021), SEAttention (Hu et al., 2018), and MHSA (Srinivas et al., 2021). As seen in Table 2, the Multi-head Self-attention Mechanism (MHSA) is introduced, which can consider multiple attention subspaces simultaneously, modeling the association relationship between different features more comprehensively and globally. This allows for better capture of the association and context information between features. In addition to having a similar mAP@0.5/% to the SimAM attention mechanism and ParNet Attention attention mechanism, MHSA, compared with other attention mechanisms, can focus on target features more accurately and improve the accuracy of target detection.

**TABLE 2:** Comparison of different attention mechanisms.

Baseline model	Attention	Parameter	FLOPs/G	mAP@0.5%
YOLOv8n + NDW + Small target detection layer	TripletAttention (Misra et al., 2020)	2983504	12.7	83.02
	CoTAttention (Li et al., 2021)	3560228	13.1	83.30
	ShuffleAttention (Yang, 2021)	2983300	12.6	83.52
	Polarized self-attention (Liu Y. et al., 2021)	3115685	12.8	83.762
	GAM_Attention (Liu Y. et al., 2021)	4622884	14.0	83.769
	GlobalContext (Cao et al., 2019)	3427649	17.7	83.823
	EffectiveSEModule (Lee and Park, 2019)	3048996	12.7	83.946
	ParNetAttention (Goyal et al., 2021)	3705892	13.2	84.104
	SimAM (Yang et al., 2021)	2983204	12.6	84.248
	SEAttention (Hu et al., 2018)	2991396	12.7	83.73
	MHSA (Srinivas et al., 2021)	3180580	12.7	84.303

To verify the effectiveness of the proposed method in this paper, we conducted comparative experiments on the smoking dataset with several mainstream object detection methods, further validating the feasibility and superiority of the improved model. The detection results are shown in Table 3. The mainstream object detection algorithms include YOLOv3-tiny (Gong et al., 2019), YOLOv4-tiny (Jiang et al., 2020), YOLOv5 (Jocher et al., 2022), YOLOv6 (Li et al., 2022), YOLOv7 (Wang C. Y. et al., 2022), YOLOX-tiny (Ge et al., 2021), SSD (Leibe et al., 2016), RetinaNet (Lin et al., 2017b) and YOLOv8, compared with our model. It can be seen that our YOLOv8-MNC training result mAP@0.5/% is higher than that of YOLOv3-tiny, YOLOv4-tiny, YOLOv5, YOLOv6, YOLOv7, YOLOX-tiny, SSD, and RetinaNet by 8.674, 15.007, 3.935, 5.987, 15.867, 6.317, 22.067, and 19.317, respectively. In this experiment, the improved YOLOv8 model, YOLOv8-MNC, achieved 85.887, which is 5.797 higher than the original YOLOv8 model. This result proves that YOLOv8-MNC is superior to other models, validating the efficiency of this model. At the same time, it also illustrates the effectiveness of our combination of NWD Loss, the multi-head self-attention mechanism (MHSA), and the use of a lightweight general-purpose upsampling operator CARAFE to replace the original nearest neighbor interpolation upsampling module. In addition, the fine-tuning of model parameters can yield more accurate and stable forecast results.

**TABLE 3:** Comparison with mainstream algorithms.

Detector	Backbone	mAP@0.5/%
YOLOv3-tiny (Gong et al., 2019)	DarkNet-53	77.213
YOLOv4-tiny (Bochkovskiy et al., 2020)	CSPDarknet53	70.88
Yolov5 (Jocher et al., 2022)	CSPDarknet53	81.952
Yolov6 (Li et al., 2022)	EfficientNet	79.90
YOLOv7 (Wang Z. et al., 2022)	CBS+E-ELAN+MP	70.02
YOLOX-tiny (Ge et al., 2021)	CSPDarknet-S	79.57
SSD (Leibe et al., 2016)	VGG16	63.82
RetinaNet (Lin et al., 2017b)	resnet50	66.57
YOLOv8	CSPDarknet53	80.09
YOLOv8-MNC	CSPDarknet53	85.887

#### 4.3.3. Ablation experiments

We proposed four improvements on the base of the YOLOv8 model: (1) introducing NDW, (2) adding MHSA attention mechanism, (3) improving the step size of the first convolution in the backbone part of the yaml file in YOLOv8, from 2 to 1, and (4) using the lightweight upsampling operator CARAFE. The improved model is evaluated using three indicators: parameters, GFLOPs, and Map0.5/%.

**FIGURE 7:**
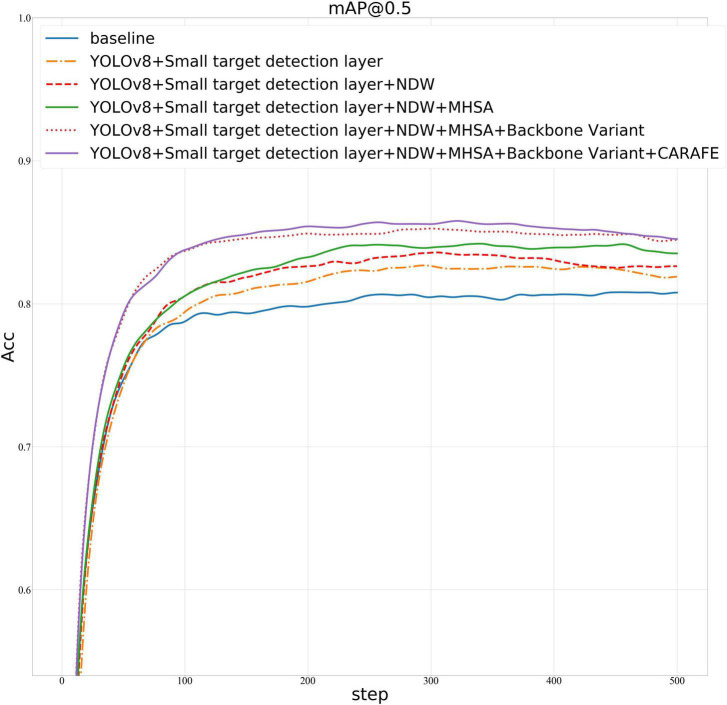
Ablation experiment line graph.

**FIGURE 8:**
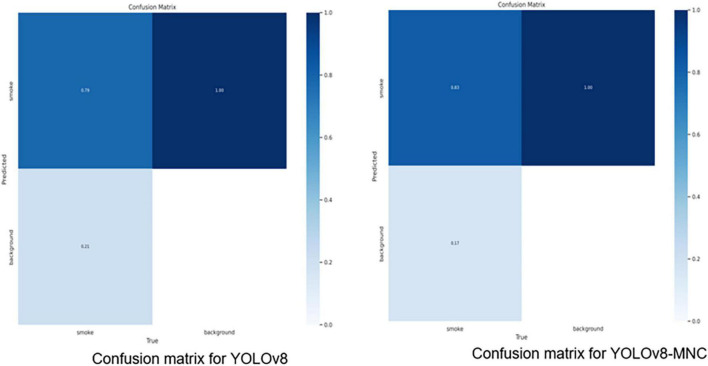
Confusion matrix diagram.

In Table 4, using the YOLOv8 model as a baseline, we introduced four key improvements to enhance its performance. The CELU activation function was adopted for its strong non-linear expression ability. A small target detection layer was added, increasing the mAP@0.5/% by 2.696. The introduction of Normalized Wasserstein Distance (NWD) further improved the mAP@0.5/% by 0.898, enhancing small target detection. The Multi-Head Attention Mechanism (MHSA) and the lightweight universal upsampling operator CARAFE contributed additional improvements. Adjusting the stride of the first convolution parameter from 2 to 1 also increased the mAP@0.5/%. The model improvement graph is shown in Figure 7. Figure 8 is the confusion matrix diagram of YOLOv8 and YOLOv8-MNC. Collectively, these enhancements led to a significant increase in mAP@0.5/%, with a notable rise in the True Positive box from 0.79 to 0.83, validating the effectiveness of the improvements and illustrating the model’s increased precision and robustness.

**TABLE 4:** Ablation experiments.

YOLOv8	Tiny object layer	NDW	MHSA	Backbone variant	CARAFE	Parameters	GFLOPs	Map0.5/%
√						3011043	8.2	80.078
√	√					2983204	12.6G	82.774
√	√	√				2983204	12.6	83.672
√	√	√	√			3180580	12.8	84.303
√	√	√	√	√		3180580	51.2	85.346
√	√	√	√	√	√	3383036	55.5	85.887

In summary, the YOLOv8-MNC algorithm outperforms other algorithms due to the following key enhancements:

(1) NWD Loss Integration: The NWD loss function reduces sensitivity to small object position deviations, enhancing training accuracy. This is achieved by normalizing IoU weights according to the target object’s size and introducing position-sensitive weights. These adjustments allow the model to predict the location and size of bounding boxes more accurately, paying more attention to details and reducing the impact of edge object position deviation.
(2) Inclusion of MHSA Attention Mechanism: The addition of the MHSA attention mechanism enables the model to better capture relationships between different locations, scales, and semantics. By computing similarities between query and key vectors, the model can focus on important regions in the image, enhancing its perception of local details and global contextual information.
(3) Stride Improvement in the Backbone Part: By changing the stride from 2 to 1 in the YOLOv8 yaml file, the model captures more detailed features and provides more location information. This adjustment allows the convolution layer to move only one pixel at a time, capturing more nuanced information.
(4) Adoption of CARAFE for Upsampling: Replacing traditional upsampling methods with CARAFE improves the spatial perception of low-resolution input images. CARAFE’s self-attention mechanism calculates from which surrounding local areas to gather information for reorganization, allowing for a more refined feature reorganization process. This ensures that the output quality matches the input, overcoming problems such as blurring and distortion in low-resolution images.

These improvements collectively contribute to the superior performance of YOLOv8-MNC, making it more sensitive and accurate in locating small targets, and enhancing its ability to process low-resolution information.

#### 4.3.4. Algorithm analysis

To further intuitively demonstrate and evaluate the test effects and compare the feature extraction capabilities of YOLOv8 and the improved YOLOv8-MNC in small target detection, we need to pay attention to what key information the main network has extracted from the pictures. In this paper, we use the more generalized Grad-CAM method to study the areas of interest of the grid output values. Grad-CAM (Gradient-weighted Class Activation Mapping), an improved version of CAM (Class Activation Mapping), uses specified class gradients to help analyze the network’s areas of interest for a particular class. By examining the network’s areas of interest, we can analyze whether the network has learned the correct features or information, making this method significantly meaningful for the visualization of image classification.

**FIGURE 9:**
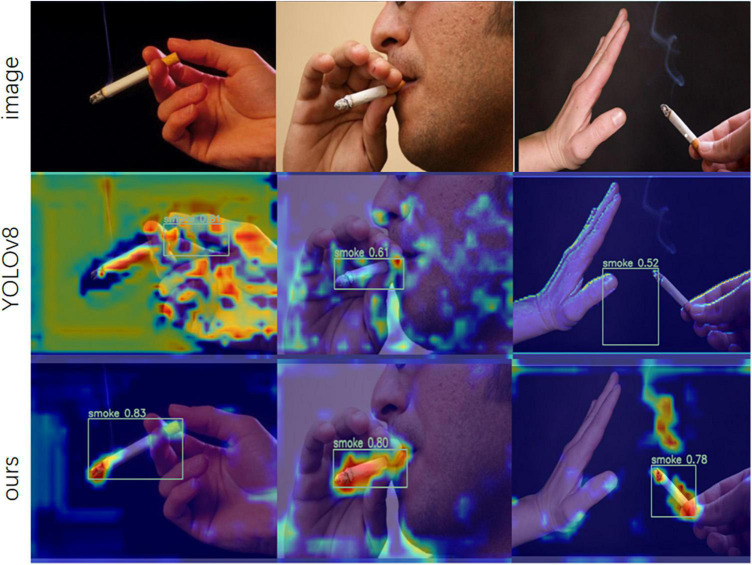
Graph of YOLOv8 and YOLOv8-MNC model test results.

Figure 9 shows the Grad-CAM images after two different networks processed the test set images. The brighter areas in the figure represent the areas the network pays more attention to. Observing the test results, it can be seen that the improved YOLOv8-MNC model covers more smoking target parts in the heat map area and is brighter and more concentrated than YOLOv8. Therefore, with the help of NWD Loss, the MHSA attention mechanism, and the lightweight upsampling operator CARAFE, the model can pay more accurate attention to the targets, reflecting the model’s efficiency and accuracy.

The performance of the smoking detection model can be challenged in real-world applications due to factors like poor visual conditions, pose and scale variations, occlusions, and real-time requirements. However, these challenges can be mitigated through strategies such as data augmentation to simulate diverse visual conditions, multi-scale training to handle scale variations, the integration of contextual or location information to manage occlusions, and model optimization to meet real-time demands. Implementing these strategies can enhance the model’s robustness and adaptability, improving its performance in various real-world scenarios.

## 5. Conclusion

This paper presents a novel smoking behavior detection model, focusing on real-time performance and accuracy, particularly in detecting small targets like cigarettes. Built upon the YOLOv8 architecture, the model introduces several enhancements. The NWD Loss is implemented to reduce sensitivity to small object position deviations, improving training accuracy. The Multi-head Self-Attention Mechanism (MHSA) is added to bolster the convolutional network’s global feature learning, and the lightweight CARAFE operator replaces the original nearest-neighbor interpolation, minimizing feature information loss during upsampling. These innovations collectively enhance both speed and accuracy. While the model demonstrates promising results on a self-made smoking dataset, its performance in real-world scenarios may be constrained by the limited diversity of the dataset. Future work should focus on collecting more varied and complex smoking datasets, reflecting a broader range of environmental factors, to further refine the model’s generalization ability in complex and dynamic environments.

## Data availability statement

The original contributions presented in this study are included in the article/supplementary material, further inquiries can be directed to the corresponding author.

## Author contributions

ZW: conceptualization, methodology, resources, data curation, and writing—review and editing. LL: software, validation, and writing—original draft preparation. PS: formal analysis and investigation. All authors have read and agreed to the published version of the manuscript.
